# Draft Genome Sequence of the Marine Bioluminescent Bacterium Aliivibrio fischeri ATCC 7744

**DOI:** 10.1128/mra.01117-21

**Published:** 2022-04-04

**Authors:** Eric Kat Jun Low, Amos Eng Liang Goh, Joko Logis, Siew Wei Lee, Ayesha Fatima, Wai Sum Yap, Crystale Siew Ying Lim

**Affiliations:** a Department of Biotechnology, Faculty of Applied Sciences, UCSI University, Wilayah Persekutuan Kuala Lumpur, Malaysia; b Beykoz Institute of Life Sciences and Biotechnology (BILSAB), Bezmialem Vakif University, Istanbul, Turkey; Montana State University

## Abstract

The Gram-negative marine bioluminescent bacterium Aliivibrio fischeri is commonly used as a bioreporter in drug inhibition studies. Its bioluminescence is regulated by the gene expression of the *luxI-luxR* quorum-sensing system. Here, we report the draft genome sequence of A. fischeri ATCC 7744, including identification of the putative *lux* operon.

## ANNOUNCEMENT

Aliivibrio fischeri ATCC 7744 is a Gram-negative, rod-shaped, marine bioluminescent bacterium that often inhabits the light organs of the Hawaiian bobtail squid (Euprymna scolopes), thus forming a natural symbiotic relationship. The expression of bioluminescence is regulated by the *luxI-luxR* quorum-sensing system, which consists of the *luxI-luxR* regulatory circuit and a *lux* operon (*luxCDABE*), in a population-density-dependent manner (1). The bioluminescence *lux* quorum-sensing mechanism of A. fischeri has been widely used in drug inhibition studies and toxicological analyses as a bioreporter (2, 3).

In this study, A. fischeri ATCC 7744 was purchased from the American Type Culture Collection (ATCC) and cultured at 26°C in BOSS medium with the addition of phosphate buffer at pH 7.2, with shaking at 150 rpm (4). Bioluminescence was observed after 18 to 24 h of cultivation in this study (BioProject accession number PRJNA669590). The genomic DNA of A. fischeri was isolated using the innuPREP DNA minikit (Analytik Jena, Germany) according to the manufacturer’s protocol. The genomic DNA library was prepared from the isolated sample using a PCR-free library preparation method that results in fragments of 270 bp and was sequenced by Codon Genomics (Malaysia) using a HiSeq 4000 system (Illumina, USA). A total of 9,344,858 paired-end 150-bp reads were generated, and their quality was assessed by FastQC v0.10.1 (5). Low-quality reads and bases (quality scores of less than Q20), ambiguous bases (Ns), and artifacts, including reads with lengths of <50 bp and reads that lost their pair during preprocessing, were filtered and removed using FASTX-Toolkit v0.0.13.2 (http://hannonlab.cshl.edu/fastx_toolkit). Approximately 98.72% clean reads (9,224,871 bp) were generated after filtering and trimming. The clean reads were assembled using the Velvet assembler v1.2.10 (6) then further improved by scaffolding and gap filling using SSPACE v2.0 (https://bioinformaticshome.com/tools/wga/descriptions/SSPACE.html#Details) and GapFiller v1.10, respectively (7, 8). Default parameters were used for all analyses unless otherwise specified.

The genome assembly of A. fischeri ATCC 7744 revealed 207 contigs (*N*_50_, 92,526 bp) totaling 4,302,243 bp in length, with a total GC content of 38.24%. Structural RNAs, including 113 tRNAs and 12 rRNAs (5S, 10; 16S, 1; 23S, 1), were predicted using tRNAscan-SE v1.3.1 (http://lowelab.ucsc.edu/tRNAscan-SE) and rRNAmmer v1.2 (https://services.healthtech.dtu.dk/service.php?RNAmmer-1.2), respectively (9, 10). The predicted tRNAs and rRNAs were masked on the genome using maskFastaFromBed of BEDtools v2.16.2 (https://bedtools.readthedocs.io/en/latest/index.html) (11). Then, a total of 3,806 protein-coding genes were predicted from the gene contents of the assembled genome using Prodigal v2.6 (12); 3,799 of the predicted protein-coding genes (≥33 amino acids) were subjected to a BLAST v2.2.28+ search against the NCBI nonredundant (NR) database and the Swiss-Prot (SP) database, which revealed that 99.0% (3,762 sequences) and 74.6% (2,834 sequences) of the peptide sequences were found in the NR and SP databases, respectively. The majority of the top species hits were from *Aliivibrio* spp. A total of 80.4% of the peptide sequences (3,055 sequences) were identified with 3,885 unique Gene Ontology (GO) identification numbers using Blast2GO v2.5.0 (13). A putative *lux* operon formed by a cluster of bioluminescence genes (*luxCDABEG* and *luxI-luxR*) was identified in Aliivibrio fischeri ATCC 7744 scaffold 1 at nucleotide positions 38976bp to 47005bp, with GO identification number 0008218.

### Data availability.

The whole-genome assembly of Aliivibrio fischeri ATCC 7744 has been deposited in NCBI GenBank under the accession number JADCNP000000000, and the project data are available under BioProject accession number PRJNA669590 and BioSample accession number SAMN16456059. The raw draft genome sequencing data have been deposited in the Sequence Read Archive (SRA) under SRA accession number SRX14356928.

## References

[1] Norsworthy AN, Visick KL. 2013. Gimme shelter: how *Vibrio fischeri* successfully navigates an animal’s multiple environments. Front Microbiol 4:356. doi:10.3389/fmicb.2013.00356. 24348467 PMC3843225

[2] Badar U, Shoeb E, Daredia K, Shawar D, Akhtar J, Ansari M. 2012. Screening and characterization of luminescent bacterial strain. J Basic Appl Sci 8:602–606.

[3] Abbas M, Adil M, Ehtisham-Ul-Haque S, Munir B, Yameen M, Ghaffar A, Shar G, Asif Tahir M, Iqbal M. 2018. *Vibrio fischeri* bioluminescence inhibition assay for ecotoxicity assessment: a review. Sci Total Environ 626:1295–1309. doi:10.1016/j.scitotenv.2018.01.066. 29898537

[4] Alves E, Faustino MA, Tomé JP, Neves MG, Tomé AC, Cavaleiro JA, Cunha Â, Gomes NC, Almeid A. 2011. Photodynamic antimicrobial chemotherapy in aquaculture: photoinactivation studies of *Vibrio fischeri*. PLoS One 6:e20970. doi:10.1371/journal.pone.0020970. 21698119 PMC3117864

[5] Andrews S. 2012. FastQC: a quality control tool for high throughput sequence data. http://www.bioinformatics.babraham.ac.uk/projects/fastqc.

[6] Zerbino DR, Birney E. 2011. Velvet: algorithms for de novo short read assembly using de Bruijn graphs. https://pubmed.ncbi.nlm.nih.gov/18349386/. 10.1101/gr.074492.107 PMC2336801 18349386

[7] Boetzer M, Henkel C, Jansen H, Butler D, Pirovano W. 2010. Scaffolding pre-assembled contigs using SSPACE. Bioinformatics 27:578–579. doi:10.1093/bioinformatics/btq683. 21149342

[8] Boetzer M, Pirovano W. 2012. Toward almost closed genomes with GapFiller. Genome Biol 13:R56. doi:10.1186/gb-2012-13-6-r56. 22731987 PMC3446322

[9] Chan PP, Lowe TM. 2019. tRNAscan-SE: searching for tRNA genes in genomic sequences. Methods Mol Biol 1962:1–14. doi:10.1007/978-1-4939-9173-0_1. 31020551 PMC6768409

[10] Lagesen K, Hallin P, Rødland E, Stærfeldt H, Rognes T, Ussery D. 2007. RNAmmer: consistent and rapid annotation of ribosomal RNA genes. Nucleic Acids Res 35:3100–3108. doi:10.1093/nar/gkm160. 17452365 PMC1888812

[11] Quinlan A, Hall I. 2010. BEDTools: a flexible suite of utilities for comparing genomic features. Bioinformatics 26:841–842. doi:10.1093/bioinformatics/btq033. 20110278 PMC2832824

[12] Tille A, Sallou O. 2014. Debian package tracker: Prodigal. https://tracker.debian.org/pkg/prodigal.

[13] Götz S, Garcia-Gomez JM, Terol J, Williams TD, Nagaraj SH, Nueda MJ, Robles M, Talon M, Dopazo J, Conesa A. 2008. High-throughput functional annotation and data mining with the Blast2GO suite. Nucleic Acids Res 36:3420–3435. doi:10.1093/nar/gkn176. 18445632 PMC2425479

